# The epistemic vices of corporations

**DOI:** 10.1007/s11229-023-04133-2

**Published:** 2023-04-20

**Authors:** Marco Meyer

**Affiliations:** Department of Philosophy, University of Hamburg, Überseering 35, Postfach #4, 22297, Hamburg, Germany

**Keywords:** Epistemic vice, Virtue epistemology, Social epistemology, Organizational epistemology, Business ethics

## Abstract

Vice epistemology studies the qualities of individuals and collectives that undermine the creation, sharing, and storing of knowledge. There is no settled understanding of which epistemic vices exist at the collective level. Yet understanding which collective epistemic vices exist is important, both to facilitate research on the antecedents and effects of collective epistemic vice, and to advance philosophical discussions such as whether some collective epistemic vices are genuinely collective. I propose an empirical approach to identifying epistemic vices in corporations, analyzing a large dataset of online employee reviews. The approach has parallels to the methodology for identifying the big-five personality traits. It surfaces epistemic vices that are attributed to corporations by its own members and reduces the number of vices to the minimum required to describe differences between corporations. This approach yields a new taxonomy of epistemic vices for corporations. While two vices identified have close correlates in the existing literature, four others have not been identified at all or only in aspects. Two of these vices are ‘genuinely’ collective in the sense that they can only be attributed to collectives.

**Supplementary Information:**

The online version contains supplementary material available at 10.1007/s11229-023-04133-2.

## Introduction

Epistemic vices are qualities of individuals or collectives that undermine the creation, sharing, and storing of knowledge (Baehr, 2011; Battaly, 2015; Cassam, 2016; 2019; de Bruin, 2015). In individuals, epistemic vices such as rigidity and indifference have been found to be associated with belief in conspiracy theories, the endorsement of fake news, and of myths about Covid-19 (Meyer, Alfano, and de Bruin, 2021).

Researchers have proposed that collectives might be epistemically virtuous or vicious in their own right (Baird & Calvard, 2019; Herzog, 2018 ch. 6; Lahroodi, 2007). This includes research that is focused on epistemic virtues and vices of organizations (Choo, 2020) and specifically of business corporations (de Bruin, 2013; Rawwas, Arjoon, and Sidani, 2013; Lamy, 2022). There has also been work investigating the epistemic virtues and vices of managers (Intezari and Pauleen, 2014; Bouilloud, Deslandes, and Mercier, 2019) and work exploring the virtues and vices that should govern truth telling between organizations (Booth and de Bruin, 2021; Koppl, 2006).

Yet there is no settled understanding of which epistemic vices exist at the collective level, or even of how to go about determining which epistemic vices collectives may possess. Collectives can vary greatly in size and structure, from a small informal group of friends to a large, complex global organization. The purpose of this article is to propose an empirical approach and apply it to one type of collective, namely business corporations. I focus on business corporations, because previous research has alleged that epistemic virtues and vices may partly explain corporate misconduct (de Bruin, 2015; Herzog, 2018; Baird and Calvard, 2019).

Current suggestions for classifying epistemic virtue and vice at the collective level are either guided by taxonomies of individual epistemic vice (Baird & Calvard, 2019) or systematically explore the ways epistemic values can be misaligned (Lamy, 2022). However, I will argue that neither of these approaches is exclusive and exhaustive (Doty and Glick, 1994). Both approaches miss epistemic vices that exist only at the collective level and hence fail to be exhaustive.

By contrast, I take an empirical approach to identifying epistemic vices of corporations that puts the collective level front and center, by analyzing a large dataset of employee reviews of the corporations they work for from the website Glassdoor.com. I use a methodology that has parallels with the methodology used to uncover the big-five personality traits (Moberg, 1999). I identify adjectives that employees commonly use in their reviews and filter this list for adjectives that relate to epistemic issues. Based on frequency counts of each adjective and words with closely related meanings, I cluster the adjectives using factor analysis, revealing six distinct epistemic vices that employees ascribe to their organizations.

The paper makes two contributions to the literature on collective epistemic vice. First, I propose a new methodology for theorizing collective vice. Second, I apply this methodology to corporate epistemic vice, yielding a new taxonomy that does not merely extend individual epistemic vice to the collective level, but puts the collective level front and center.

The taxonomy is of philosophical interest, in three ways. First, only two of the six vices identified have close correlates in the existing literature: “Incompetence” and “Dishonesty”. Four others have not been identified at all or only in aspects: “Unreliability”, “Fragmentation”, “Ambiguity”, and “Complexity”. It is noteworthy that half of the epistemic virtues identified are “faculty-like”, as are perception or memory in the individual case (Sosa, 1985), rather than “trait-like”, as are open-mindedness or curiosity (Zagzebski, 1996). Second, the last two of these vices exist exclusively at the collective level, providing examples of genuinely collective epistemic vice, in the sense that they can only be attributed to collectives, not individuals. Third, I provide evidence that employees perceive epistemic vice to emerge in three ways: as a result of the accumulated vices of their members – and in particular, of the management in the organization; as the product of the interactions among their members in the collective’s structure and culture; and as an emergent phenomenon that only exists at the level of the collective (de Ridder, 2022).

The reminder of the article is structured as follows. After introducing the concept of collective epistemic vice, I review two taxonomies of collective epistemic vice, and argue that they fail to meet the conditions of being exhaustive and exclusive. Then I describe an alternative, empirical methodology for classifying collective epistemic vice that draws on key features of the methodology that personality psychologists used to identify the five factor model of personality. This non-technical overview of the method identifies the driving assumptions of my methodology and explains how it meets the desiderata. The following section shows the technical detail of applying the method. Finally, I discuss the philosophical implications of this empirical exercise.

## Taxonomies of collective epistemic vice

Epistemic vices are the mirror-image of epistemic virtues: While epistemic vices undermine the creation, sharing, and storing of knowledge, epistemic virtues support these activities (Kidd, Battaly, and Cassam, 2020). Collective epistemic vice refers to qualities of collectives that undermine the creation, sharing, and storing of knowledge. This raises the question of how individual and collective epistemic vices are related (Lackey, 2021). Summative approaches evaluate the epistemic properties of a group based on the properties of its individual members. In this view, a group is epistemically virtuous to the extent that its individual members possess epistemic virtues, such as open-mindedness, critical thinking, and intellectual humility. Conversely, a group is epistemically vicious to the extent that its individual members possess epistemic vices, such as closed-mindedness, dogmatism, or intellectual arrogance.

Most philosophers accept non-summative accounts of epistemic virtue and vice (Byerly, 2022). This view is supported by examples of groups exhibiting traits that their members do not display (Mejia and Skorburg, 2022; List and Pettit, 2011; Lahroodi, 2019). These examples often involve group policies, procedures, or organizational culture that regulate members’ behavior when acting as a group (Miller, 2010; Bratman, 2022). These features at the collective level either lead members to behave differently when they are in the group context, or lead to emergent characteristics at the collective level that cannot be reduced to the individual level. For example, a group that is characterized by productive disagreement, mutual respect, and robust deliberation might be considered to possess an emergent property of epistemic virtue, even if some of its individual members possess epistemic vices. Conversely, a group that is characterized by intellectual conformity, deference to authority, or exclusionary practices might be considered to possess an emergent property of epistemic vice, even if some of its individual members possess epistemic virtues (Lefevere and Schliesser, 2014). Hence, the collective may display a certain range of virtue and vices, even if individual members do not share those same qualities as private individuals (Bland, 2022; Astola, 2021).

Do collective epistemic vices require vicious intent (Tanesini, 2018)? Miranda Fricker (2010) maintains that collective faculty vices are characterized by the lack of a commitment of the members of the collective to achieve epistemic goals sufficiently reliably, hence requiring no motivational component. This faculty-based approach to thinking about epistemic vices is rooted in the virtue reliabilists tradition, which has focused on virtues that are faculties such as perception or memory in the case of individuals (Sosa, 1985). By contrast, Fricker maintains that collective character vices require a joint commitment by the members of the collective to achieve a bad motive. The trait-based approach is rooted in the virtue responsibilist tradition, focussed on virtues that are cultivated traits such as curiosity or humility (Zagzebski, 1996; Montmarquet, 1993; Roberts and Wood, 2007). Many virtue responsibilists see motivation as a key requirement of epistemic virtues, yet some reject the requirement (Cassam, 2019). Carter and Broncano-Berrocal (Broncano-Berrocal and Carter, 2021, ch. 8) argue that for collectives to have trait vices, they need not be oriented towards bad epistemic motives. Rather, it is sufficient if they lack a commitment towards epistemically good motives. The reason is that the requirement of bad motive excludes plausible collective character vices such as intellectual laziness or intellectual cowardice.

We should allow that excellent cognition requires both faculty-virtues and trait-virtues (Fleisher, 2017). Similarly, deficient cognition can be due to deficient faculties as well as poorly cultivated traits. I use the term “qualities” to refer to both faculty-vices and trait-vices. A motivation to achieve a bad epistemic end may be part of some collective vices, such as epistemic malevolence (Baehr, 2010), but is not required of all epistemic vices.

Boudewijn de Bruin and Barend de Rooij’s (2022) non-summative approach to collective epistemic vice is particularly instructive for the present study. The authors propose a functionalist approach to collective virtue and vice, which attributes epistemic virtue to collectives if they are structured in a way that allows them to function as if they were virtuous agents. According to this view, an organization that is epistemically virtuous is made up of three components: it provides support for virtuous behavior at an organizational level, it has remedies in place to prevent vice, and it selects individuals who exhibit the virtues required to perform the organization’s functions. When organizations fail to create a corporate structure that is virtuous in this way, they display collective epistemic vice. This approach resonates with the results of this study, because the epistemic vices identified can be seen as failures to support epistemically virtuous behavior, or failures to remedy epistemic challenges that arise in organizations.

I maintain that collectives can display faculty-vices, because they can develop structures and systems that function like cognitive faculties in individuals (Hormio, 2018). For instance, filing systems allow the storage and sharing of information in organizations. The quality of such systems can range from excellent to deficient in supporting members of the organization in dealing with information. Monitoring and reporting processes can be seen as faculties for generating knowledge. An organizational system, process, or governance structure can therefore be epistemically vicious if it undermines the generation, sharing, or storing of knowledge. As a result, an organization can be epistemically vicious in virtue of operating such a system, process, or governance structure.^1^

Moreover, I maintain that collectives can display trait-vices, which involve the absence of an organizational commitment to good epistemic motives, or even the outright endorsement of bad epistemic motives. De Ridder (2022) has systematized three ways in which epistemic virtues and vices, including particular trait-virtues and trait-vices, can be attributed to collectives. First, collectives can be epistemically vicious in an ‘additive fashion’ if all or most of its members are epistemically vicious (de Bruin, 2015). Second, ‘interaction’ due to the collective’s governing structure or culture can generate a disposition to epistemically vicious behavior (Dempsey, 2015; Miller, 2007). Third, collectives can display ‘emergent’ collective epistemic virtues and vices, i.e., virtues and vices that only collectives can possess, such as solidarity (Battaly, 2022).

Collective epistemic vices are typically seen as negative because they undermine organizational functioning (Alvesson and Spicer, 2012) or lead to unethical decision-making in organizations (de Bruin, 2015). Yet epistemic virtue can also be problematic, if its ‘acquisitionist’ focus leads to a lack of ‘epistemic restraint’.^2^ For instance, curious organizations may develop a tendency to surveil customers (Zuboff, 2019; Manson, 2012). This information may be used to control and exploit rather than benefit customers. Hence epistemic virtue does by no means guarantee ethical behavior. The focus of this paper is to develop a taxonomy of corporate epistemic vice. It is a further question whether there are organizational behaviors associated with epistemically vicious organizations, and if so what the ethical status of each of these behaviors is.

There are several proposals but no settled understanding of which epistemic vices exist at the collective level. Previous research has focused on specific collective epistemic virtues and vices, such as epistemic injustice (Herzog, 2018, ch. 6), solidarity (Battaly, 2022), humility (Frostenson, 2016), gullibility (Laroche et al., 2019), and truthfulness (Radoilska, 2008). However, these investigations into specific vices are not intended to yield a comprehensive taxonomy.

Such a taxonomy should meet two desiderata (Doty and Glick, 1994): First, it should provide a scheme to classify epistemically vicious characteristics of corporations into mutually exclusive vices, i.e. there should not be conceptual overlap between the stipulated epistemic vices. Second, the stipulated epistemic vices should be exhaustive, i.e. there should be an epistemic vice for each epistemic deficiencies of corporations. Regarding the latter criterion, I highlight that a taxonomy should focus on the common epistemic deficiencies of corporations, rather than all conceivable deficiencies.

Baird and Calvard (2019) have suggested a taxonomy of collective epistemic vice. Like my approach, they do not tackle collectives in general, but focus on a particular type of collective, namely organizations. They distinguish four epistemic vices widely discussed at the individual level which are, in their view, involved in many cases of organizational wrongdoing: (1) Epistemic malevolence is the willful opposition to the epistemic good of others, and often issues in acts of deception, obfuscation, or hiding of knowledge (Baehr, 2010). (2) Epistemic insouciance consists in an indifference towards epistemic goods, which manifests itself in uncritically maintaining one’s beliefs in the light of contrary evidence (Cassam, 2018). (3) Epistemic hubris is an inflated sense of epistemic privilege and pride (Tanesini, 2016). (4) Epistemic injustice consists in wronging others in terms of their credibility and capacity as a knower, for instance by taking the contributions by women less seriously than those of men (Fricker, 2007).

Baird and Calvard’s taxonomy illustrate each of these vices with examples of organizations and point out their devastating consequences. Yet their taxonomy is primarily informed by the academic literature on individual epistemic vice. This approach fits the authors’ primary objective to demonstrate the relevance of epistemic vice for organizational wrongdoing. However, the approach does not provide an exclusive and exhaustive taxonomy – nor do the authors maintain otherwise. Their approach risks focusing on vices that have been highlighted in the philosophical discussion of individual epistemic vice, rather than because of their importance for understanding epistemic issues in a real-world setting. For instance, structured collectives such as organizations are often characterized by division of labor and hierarchy (Child, 2019). These features of organizations lead to characteristic epistemic challenges, including issues of knowledge management (Choo, 2006) and speak up (Burris et al., 2013). Some of the epistemic vices listed by Baird and Calvard are relevant to these epistemic challenges of organizations. Yet the methodology of importing epistemic vices identified at the individual level to the collective level does not ensure exhaustive coverage. The reason is that the specific epistemic challenges that organizations face, including division of labor and hierarchy, may well not be readily addressed by any individual-type vice.

Lamy (2022) takes a more systematic approach to classifying epistemic virtues and vices of businesses. His approach is based on the notion of the epistemic value of a message. He contends that messages can have eight different types of value, including value as the evidence the recipient requires to believe a message, value as the evidence the sender believes she must produce to meet the needs of the recipient, and value as the evidence actually received by the recipient. Epistemic vice consists in dispositions to misalign any two of these eight types of epistemic value. For instance, Lamy analyzes the vice of epistemic injustice as the disposition of the recipient to perceive the epistemic value of a message to be lower than the received epistemic value.

Lamy’s taxonomy is helpful in providing a systematic way of describing epistemic vices, if focused on individuals, not collectives. However, his approach offers little guidance on how to identify epistemic vices that are relevant in a collective setting. There are 112 different ways in which any two epistemic values can be misaligned. This complex taxonomy fails to focus attention on the most important epistemic vices in collectives. Moreover, similarly to Baird and Calvard’s approach, Lamy’s taxonomy of epistemic value abstracts from the specific epistemic challenges in collective settings, including division of labor and hierarchy. As a result, despite its complexity, the taxonomy risks not being exhaustive.

To my knowledge, there is not a taxonomy of collective epistemic vice that is exhaustive and exclusive, at least for a certain type of collective. Yet meeting these criteria is important, both to facilitate research on the antecedents and effects of collective epistemic vice, and to advance philosophical discussions on collective epistemic vice. In the following sections, I propose and apply a methodology that meets these criteria for one type of collective, namely corporations.

## An empirical approach to identifying epistemic vices of corporations

In this section, I describe, in a non-technical manner, the main elements of an alternative methodology to classify organizational epistemic vices. The methodology draws on central features of the methodology that psychologists used to identify the five-factor model personality. The five-factor model is the standard model for describing the personality traits of adults (Corr and Matthews, 2020). Most readers will be familiar with its factors: open-mindedness, conscientiousness, extroversion, agreeableness, and neuroticism. Allowing for slight differences, studies have found the same five factors regardless of age, gender, language, and cultural background of participants (Robins, Fraley, and Krueger, 2009). Moreover, the big five personality traits have been shown to be systematically related to a broad range of behaviors and achievements, including risk taking (Nicholson et al., 2005), household finances (Brown and Taylor, 2014), and job performance (Barrick & Mount, 1991).

The first step in developing the five-factor model was to identify a long-list of personality traits. To achieve this goal, the architects of the five-factor model relied on the ‘lexical hypothesis’ – the hypothesis that single words in ordinary language encode all personality differences (Moberg, 1999). Allport and Odbert (1936) identified more than 4.000 words in the English language that can be used to describe personality traits (e.g. “clever”, “gregarious”, “dependable”). This list, together with other related work, became the starting point for the development of the five factor model. The big idea is that if the ‘lexical hypothesis’ is correct, starting from an exhaustive list of trait words ensures that no possible personality trait gets missed. This approach is therefore an important step to ensure the salience of the resulting taxonomy.

I put the ‘lexical hypothesis’ to work to identify potential organizational epistemic vices. Rather than starting from all the adjectives in the dictionary, I extract all adjectives commonly used by employees in their reviews of the companies they work for. In a manual step, I select adjectives relating to epistemic issues, resulting in a list of 71 adjectives that potentially encode epistemic vices in organizations.

The second step in developing the five factor model was to quantify the extent to which people possess each of the candidate personality traits expressed by the trait words identified in the previous step. The fundamental methodological assumption that psychologists relied on to make these traits measurable is that character traits can be identified ‘democratically’, i.e., the snap judgments of ordinary people are good guides to whether they or others possess certain character traits (Moberg, 1999). It is worth emphasizing how different this approach is to the approach that virtue ethicists from Plato to Aquinas to Hume have taken, which can be seen as precursors to modern personality psychology (Corr and Matthews, 2020). They relied on theoretical reflections to systematize virtues. By contrast, the ‘democratic’ assumption led psychologists to construct questionnaires, turning each candidate trait word into a statement that respondents could agree or disagree with as descriptions of themselves or others, depending on the focus of the study. For instance, in a seminal study Tupes and Christal (1958) asked Air Force officers to rate their colleagues on a large number of personality traits. Note that the theoretical approach, which still prevails concerning collective epistemic vices, neither led to the convergence on one framework nor to instruments with the explanatory and predictive power that the five factor model based on the ‘democratic assumption’ achieved.

I adopt the ‘democratic’ assumption by basing my analysis of the epistemic vices of organizations on the reviews employees leave of the organizations they work for on the online portal Glassdoor.com. In contrast to the research on the five-factor model, I do not develop a survey that employees need to complete. Rather, I use a methodology from computational linguistics, analyzing large amounts of text produced for a different purpose about the downsides of working for their organizations (Campbell and Shang, 2021). I search the written comments for the trait words identified in the first step, as well as words with a very similar meaning in the context of the dataset, using a methodology further detailed in the next section. Counting words with similar meanings in addition to the trait words helps to broaden the database, making the resulting analysis more robust to random fluctuations in the use of a single term in reviews of a specific company. The result of this step is a table with one row per company, and one column for each of the trait words. The cells record how often each trait word, and the words similar in meaning, occur in the reviews pertaining to the respective company.

The third step in developing the five factor model is to reduce the number of personality traits to the minimal number required to describe psychological differences in character between people. The result of this step is the clustering of personality traits in the five familiar clusters that we now use to describe differences in personality. The challenge is to reduce the large number of personality traits into a model of mutually exclusive types. Psychologists wanted to find the minimum number of mutually exclusive personality traits needed to account for the vast number of differences between people. “Gregarious” and “sociable” are two trait words with subtly different meanings. But most people who are gregarious tend to be sociable as well, and vice versa. Hence a model of character should not treat these personality traits as separate. To identify a simple model of mutually exclusive traits, psychologists relied on the method of factor analysis (DeVellis, 2016). Factor analysis identifies patterns in datasets and can be used to find groups of personality traits that respondents consistently ascribe jointly to individuals. The famous five factors of personality are simply the names researchers gave to the groups of items that clustered together in studies attempting to reduce a longer list of personality traits to a simple model using factor analysis.

To identify a model of mutually exclusive epistemic vices of corporations, I also employ the method of factor analysis. My analysis is based on the table that resulted from the previous step, describing how often trait words and their close substitutes are used to describe each company in the dataset. The factor analysis suggests that trait words can usefully be grouped according to seven clusters. For a given company, mentions of words in the same cluster are likely to be more similar than mentions of words belonging to different factors. I manually label each of the resulting groups of trait words to capture the core of their meaning. I will discuss the characteristics of these factors and how they relate to the existing taxonomies of epistemic vice in the discussion section.

I want to highlight how this approach addresses the shortcomings of existing taxonomies outlined in the previous section. In contrast to existing taxonomies, this methodology ensures that the resulting taxonomy is exhaustive, in the sense that it picks up all the epistemic issues that employees discuss with regard to business corporations. If we accept the ‘democratic’ hypothesis, the complaints of employees provide an excellent starting point for identifying the vices that undermine the creation, sharing, and storing of knowledge in corporations. The methodology is not prejudiced to identify epistemic vices that have been posited on the individual level, and is able to identify vices that are only relevant on the collective level. Moreover, the methodology ensures that the resulting types are mutually exclusive, because it clusters potential epistemic vices in such a way that the resulting clusters do not overlap and account for actual differences between organizations.

While the proposed methodology has an empirical core, it is still informed (and influenced) by theory. While factor analysis reveals word clusters, clusters still have to be named and the underlying concepts defined. Naming and defining clusters allows to connect the results to the existing literature, but also introduces discretion. I used this discretion to compare and contrast resulting clusters with existing theoretical constructs, aiming to connect the resulting clusters to existing constructs where possible. Cluster names and definitions emerge in an iterative process between considering data and existing theory.

## Empirical analysis

In this section, I apply the methodology described in the previous section to a large dataset of online employee reviews of US corporations, resulting in a new taxonomy of corporate vice. The social media platform Glassdoor provided me with access to a snapshot of reviews collected on their website. Glassdoor is a platform where employees review and reflect their experience with related firms anonymously. Individuals can post reviews about their current and former employer(s). Glassdoor does not systematically verify the employment status of reviewers, but requires users to either register a valid company email address or provide a valid social networking account. This data is used to identify and remove suspicious users. Each individual is allowed one active review per employer, per year. If a user updates a review within a year, only the newer review will be listed. Glassdoor’s community standards encourage reviewers to comment on employers where they have held jobs “within the last five years so it’s relevant to today’s job seeker” (Glassdoor, 2022). Individuals can also review companies they have only interviewed for when focussing on the recruitment process in their reviews. Users are encouraged to provide a balanced review, discussing both the pros and the cons of an employer. Glassdoor screens reviews for safety and reliability, removing reviews that contain negative remarks about identifiable individuals outside the senior leadership of a company, threats of violence, discriminatory language, or reveal confidential information. However, the Community Guidelines state that Glassdoor expects users to provide truthful comments, and does not “take sides when it comes to factual disputes” (Glassdoor, 2022).

Glassdoor provided 1,3 million reviews of 868 US companies collected between 2008 and 2020 that have been reviewed at least 100 times. As a result, the companies in the dataset tend to be large. In addition to quantitative judgments about the work experience at the company, employees also write down their comments regarding their positive and negative experience with their companies. The negative comments left in these reviews are the basis for analysis. The large number of reviews pertaining to a large number of different corporations make the dataset an excellent candidate for applying the methodology.

### Identifying word clusters related to epistemic vice

The first task is to identify the parts of the reviews where employees ascribe epistemic vice to the corporations they work for. To be more precise, reviews characterize entities at different levels, including the corporation as a whole (e.g. “the company is still a little disorganized”), processes and systems within the company (e.g. “byzantine performance review system”), and functional groups such as management (“constantly contradictory statements from management”). As a shorthand, we treat any of these ascriptions as pertaining to the corporation.^3^

As discussed in the previous section, I rely on the ‘lexical hypothesis’, according to which single words in ordinary language encode all vice ascriptions. To identify the relevant words, I extract all adjectives used in the reviews and lemmatize them, so that e.g. the comparative and superlative forms of adjectives (e.g. “bigger”, “biggest”) are not counted separately from their positive forms (“big”). This results in 960 adjectives that occur at least once in every 10.000 reviews. I and two other researchers working on social epistemology in organizations manually label these adjectives independently. We classified each adjective depending on whether (1) it is regularly used to characterize the way an organization deals with information and (2) it has a negative connotation. Examples that all of us selected are “stupid”, “disorganized”, and “rigid”. Each of us discarded for instance “stressful”, “large”, and “honest”. Our independent classifications results in an inter-rater agreement of 0,71 (Fleiss’ Kappa), suggesting “good” agreement (0,6 − 0,75) (Cicchetti & Sparrow, 1981). The result is a list of 82 adjectives. The list of selected adjectives is part of the Supplementary Information (Table S2).

Rather than relying on individual trait words alone, I create word clusters for each trait word based on the most similar word embeddings to the trait word using a Word2Vec model based on the dataset of employee reviews (Rong, 2016). The reason is to base the analysis on the meaning of the trait words, rather than mere syntactic word representations. As Kowsari et al. (2019, 7) explain, the words “airplane”, “aeroplane”, “plane”, and “aircraft” are often used interchangeably, yet would be counted separately if we relied on syntactic word representations. Moreover, capturing semantically closely related words is important for our analysis to ensure that no individual review has too much sway over the outcome. This step is necessary because the least common trait word selected, “unstructured”, occurs only 325 times across all reviews – on average less than once for each company in our dataset. As a result, whether just a single reviewer mentions a trait word would have considerable influence on a company’s score for a particular trait word. Yet it is preferable if a company’s score on any one epistemic vice is not overly reliant on single reviews by employees.

To make epistemic vice scores less dependent on single mentions of a single word, I rely on the similarity of word vectors based on word embeddings, a common feature engineering technique in text classification tasks (Kowsari et al., 2019, 7). Specifically, for each trait word, I identify the five words most similar in meaning, and add their counts to the count of the trait word. For instance, for the trait word “unrealistic”, I identify “unreasonable”, “unreachable”, “unattainable”, “unachievable”, and “unobtainable”. To find these similar words, I compute word embeddings for each of the trait words using a Word2Vec model, a neural-network based learning algorithm (Rong, 2016). Word embeddings are low-dimensional numerical representations of a word in a given corpus to quantify semantic similarity. Using the method described by Elekes et al. (2017), I use the word vectors of the trait words identified in the previous step to identify the five words in the dataset with the most similar word vector. Two words are semantically similar if the vectors representing them are close according to the cosine similarity distance function. It is worth emphasizing that the Word2Vec model is trained on the Glassdoor dataset, rather than a general English-language corpus. For this reason, the model is able to identify close substitutes in meaning also for words that acquire unusual meanings in a corporate context. For instance, the model identifies “bureaucratic” as similar in meaning to “political”, thus picking up on the tendency in corporations to use the term “political” as an antonym to technical or professional competence, rather than as a synonym to “civic” or “official”. Moreover, the model is also able to identify common misspellings in the dataset, such as “beaucratic” and “beauracratic”.

The outcome of this procedure is a word cluster for each of the 82 trait words. These word clusters occur more frequently in the dataset, making the analysis more robust to views of single respondents. Words from the least common word cluster, “oppressive”, occur 836 times in the dataset, or three times more frequently than the trait word alone, and roughly once per company. For a full list of trait words and the five words similar in meaning, refer to Table S2 in the Supplementary Information.

### Measuring ascriptions of word clusters to companies

The second task to quantify to what extent employees ascribe each of the traits captured by a trait word cluster to each company in the dataset. To do this, I lemmatize each review, and count, for each company, the number of times one of the words in each word cluster occurs. The result of this step is a table with one row per company, and one column per word cluster. The cells contain the number of times words from the respective word cluster occur in the reviews of the respective company. Table S2 in the Supplementary Information shows summary statistics for each of the word clusters. Mentions range from 836 to 18.828. Mentions are correlated, to varying degrees. Correlations of mentions between companies range from − 0,17 to + 0,99. Average correlations between mentions are positive for all word clusters, with an average of 0,05. These correlations indicate that if a company scores high on one trait word cluster, it is on average slightly more likely to score high on others. Yet there is a lot of variance in the extent to which word clusters correlate. For some combinations of word clusters, correlations are even negative.

### Reducing dimensionality via factor analysis

The third task consists in conducting a factor analysis to identify a simple model of mutually exclusive traits. The last step resulted in a table with one row per company, and one column for each of the 82 word clusters, capturing how often words from each trait word cluster are mentioned per company. The Kaiser-Meyer-Olkin index (KMO) for the dataset is 0,68, indicating “middling” sampling adequacy (Kaiser, 1974) – which suggests that factor analysis is an adequate methodology to apply for this dataset. Factor analysis essentially helps to simplify this table with 82 columns – one column per word cluster – to a smaller number of columns. Mathematically, this is achieved by creating a smaller number of new columns, called factors, which best condense the information contained in the 82 original columns. Since a factor model is an approximation, researchers face a tradeoff between simplicity, suggesting the extraction of fewer factors, and accuracy, suggesting more factors. Each additional factor explains a smaller additional proportion of the underlying variance in the data. A scree plot can be used to determine the number of factors to retain in a factor analysis. Scree plots display the eigenvalues, a measure for the amount of additional variance explained, in a downward curve. Cattell (1966) suggests retaining factors to the left of the “elbow” of the graph where the eigenvalues level off. The scree test suggests retaining six factors (see Figure S2 in the Supplementary Information).

I conduct the factor analysis as an iterative process. In each round of analysis, I extract six factors. I use an oblique rotation (oblimin), which allows for the resulting factors to be correlated (Peterson, 2017). The first round of factor analysis is applied to all 82 word clusters. The results, however, do not meet psychometric standards for an exploratory factor analysis (DeVellis, 2016), calling for further rounds of factor analysis on a subset of word clusters once problematic clusters have been excluded. For detailed results of the initial factor analysis, see Table S3 in the Supplementary Information. To find word clusters that can be factorized, I apply two criteria. First, I eliminate word clusters whose loadings on none of the factors is greater than a commonly used cut-off point of 0,4 (DeVellis, 2016). Second, I eliminate word clusters with large cross-loadings, defined on loadings on the primary factor that are not at least 0,3 larger than the next-biggest loading (DeVellis, 2016). One reason why large cross-loadings on other factors occur is that the words involved capture epistemic vice in general, rather than any specific aspect of it (e.g. “stupid”, “incorrect”, “vague”). To obtain factors of word clusters that focus on specific organizational epistemic vices, I exclude the word clusters identified as problematic from further rounds of factor analysis.

After applying this procedure three times, all 31 remaining word clusters meet the required threshold. The results are presented in Table 1. The KMO for the dataset is 0,79, indicating “middling” sampling adequacy, making factor analysis appropriate. The factors capture 69% of the variance in the dataset. This final factor analysis meets psychometric criteria for an exploratory factor analysis.

**Table 1:** Results of the final factor analysis extracting six factors from counts per company of 31 word clusters

Word cluster	Incompetence	Unreliability	Fragmentation	Ambiguity	Complexity	Dishonesty
incompetent_cluster	0,92	0,00	-0,02	-0,01	-0,05	0,01
inept_cluster	0,91	0,00	0,01	0,02	0,04	-0,01
incapable_cluster	0,87	-0,01	0,01	-0,03	0,04	0,01
unqualified_cluster	0,83	0,03	0,00	-0,01	-0,06	-0,01
clueless_cluster	0,83	-0,01	0,01	0,02	0,06	-0,02
inexperienced_cluster	0,83	0,02	0,01	0,00	-0,05	-0,03
ignorant_cluster	0,76	-0,03	-0,05	-0,05	-0,02	0,07
ineffective_cluster	0,74	0,01	0,07	0,06	0,11	0,00
lazy_cluster	0,71	-0,03	-0,05	0,00	-0,07	-0,01
unstructured_cluster	0,01	1,00	0,00	0,01	-0,01	0,00
disorganized_cluster	0,00	1,00	0,00	-0,01	0,00	0,00
chaotic_cluster	0,00	1,00	0,00	0,01	0,00	0,00
unorganized_cluster	0,01	0,98	0,00	-0,01	0,01	0,00
sloppy_cluster	0,00	0,58	0,00	0,04	0,03	-0,01
unreliable_cluster	0,01	0,54	-0,04	-0,04	-0,01	-0,02
bureaucratic_cluster	0,01	0,00	0,97	0,00	-0,02	0,00
matrixed_cluster	0,00	0,04	0,90	-0,01	0,01	0,00
hierarchical_cluster	0,02	-0,01	0,86	-0,02	0,00	0,01
political_cluster	0,11	-0,11	0,56	0,08	0,02	0,00
ambiguous_cluster	0,01	0,01	0,00	0,97	-0,01	-0,02
vague_cluster	0,00	-0,01	0,00	0,97	0,00	-0,01
conflicting_cluster	0,03	0,00	0,00	0,69	0,01	0,05
contradictory_cluster	0,04	-0,02	0,01	0,52	0,05	0,07
complicated_cluster	0,02	0,00	-0,01	-0,02	0,99	-0,01
convoluted_cluster	0,02	0,03	-0,03	0,02	0,82	0,04
complex_cluster	0,03	0,01	0,08	-0,03	0,71	-0,05
confusing_cluster	0,02	-0,04	-0,04	0,04	0,67	0,02
manipulative_cluster	0,00	0,01	0,01	-0,01	-0,01	0,98
untrustworthy_cluster	0,00	0,01	0,01	-0,01	-0,01	0,98
dishonest_cluster	0,02	-0,01	-0,04	0,03	0,07	0,57
deceptive_cluster	0,01	-0,05	-0,07	0,04	0,02	0,56

Judging by which word clusters load on the same factor, the results seem plausible at face value, e.g., “manipulative”, “untrustworthy”, “dishonest” and “deceptive” load on the same factor. To further verify the distinguishing features of each factor, I read through several hundred example sentences mapped to each factor. Based on these data points, I named each cluster and developed a definition for each factor. In naming clusters, I stayed close to the words used by employees. With the exception of the label “fragmentation”, I used the substantiation of a common adjective in the word cloud. “Fragmented” is not part of the respective word cloud, but strikes me as capturing better than any of the adjectives contained in the word cloud the underlying epistemic challenge of “bureaucratic”, “matrixed”, “political” and “hierarchical” organizations: That a fragmented organizational structure or interdepartmental dynamics undermine the creation or sharing of organizational knowledge. Table 2 summarizes the results and gives examples of comments for each of the corporate vices identified.

**Table 2:** Definition of epistemic vices based on factor analysis, including the relevant trait words, the frequency of trait word cluster words occurring in the dataset, and manually selected example phrases pertaining to each factor

Epistemic Vice	Word clusters	Examples
*Incompetence* People in the organization lack the competence to do their job well.	arrogant, uninvolved, ineffective, unmotivated, impotent, capable, oblivious, underqualified, incompetent, bossy, untrained, unsuited, spineless, inexperienced, incompetant, unaware, weak, nosey, inept, clueless, ignorant, uneducated, unexperienced, lazy, inefficient, unintelligent, disrespectful, incapable, unqualified, ineffectual	“Directors […] sit in their offices upstairs, completely unaware of what is going on […].” “Young and inexperienced work force is not capable of executing senior management’s concept of a winning corporate strategy.” “Management is either so incompetent or fearful for their own jobs that they cannot possibly make good decisions.”
*Unreliability* The organization lacks reliable mechanisms for the generation, sharing, or storing of organizational knowledge.	unstructured, faulty, unorganised, sporadic, disorganized, disorganised, unorganized, haphazard, amateurish, shoddy, undependable, messy, flakey, frenetic, unreliable, unfocused, uncoordinated, sloppy, chaotic	“undependable work systems” “The company is still a little disorganized from growing so quickly. Sometimes the informational paths haven’t been established yet to make sure everyone knows what they need to.” “Management is extremely disorganized and doesn’t communicate.”
*Fragmentation* Fragmented organizational structure or interdepartmental dynamics undermine the creation or sharing of organizational knowledge.	bureaucratic, decentralized, matrixed, beaucratic, beurocratic, matrix, layered, political, beaurocratic, hierarchial, hierarchical, hierarchal, hierarchy, politicized, bureacratic, politcal, beauracratic, heirarchy	“Very bureaucratic, never listen to people below managers and leaders.” “Sometimes the simplest changes take a very long time to implement within the matrix structure.” “Two many layers of organization. Decision makers are too far up the ladder to have an audience with. Clear communication is often hindered by the hierarchy.”
*Ambiguity* Role conflict and ambiguity undermine the creation and sharing of organizational knowledge.	contradict, contradicting, nebulous, unclear, ambiguous, directions, unorthodox, contradictory, undefined, vague, conflicting, definitions, differing	“Constantly contradictory statements from management” “Management of Sales, Service, Accounting and Plant Operations are not on the same page…and often have conflicting goals.” “Unclear and changing strategy, uncertainity on next steps, Sr. Leaders not very inspiring”
*Complexity* Complex or otherwise inefficient processes undermine the creation, sharing, or storing of organizational knowledge.	convoluted, matrixed, inefficient, byzantine, unwieldy, frustrating, confusing, complicated, intricate, cumbersome, complex, unintuitive, jumbled	“Byzantine performance review system, which allows a lot of room for subjectivity and effectively discourages team work and collaboration with its stack ranking.” “Overly complex internal processes force you to spend too much time on non value added tasks.” “[Company] is always starting new directives and plans many of which fail or are rolled out very poorly and this can be very frustrating at the store level.”
*Dishonesty* The organization has a tendency to lie, deceive, or mislead.	deceitful, unethical, deceptive, unscrupulous, manipulative, dishonest, conniving, deceiving, misleading, shady, vindictive, untrustworthy	“These folks have been the most dishonest, underhanded bunch that I’ve dealt with in over 30 years.” “at times deceptive selling tactics by other employees.” “Shady business practices at individually owned branches.”

According to de Bruin and de Rooij’s functionalist account of epistemic vice introduced above, the six qualities of organizations identified by the taxonomy are epistemic vices, because they characterize organizations that fail to provide support for epistemically virtuous behavior at an organizational level (“Unreliability”, “Ambiguity”); lacks remedies to prevent epistemically vicious behavior (“Complexity”, “Fragmentations”; and fails to select individuals who exhibit the virtues required to perform the organization’s function (“Incompetence”, “Dishonesty”).

Some of the selected quotes explicitly charge the respective corporation with epistemic vice (Kidd, 2018). Consider for instance one of the examples given for the vice of “Unreliability”: “The company is still a little disorganized from growing so quickly. Sometimes the informational paths haven’t been established yet to make sure everyone knows what they need to.” The author of the review names a mechanism (“informational paths have not been established”) and links this mechanism to a bad epistemic outcome (some people do not “know what they need to”). I consider examples that follow the definition of the vice closely explicit epistemic vice charges.

But note that many of the example sentences do not contain explicit epistemic vice charges. For instance, consider this example given for the vice of “Ambiguity”: “Constantly contradictory statements from management.” This statement contains an observation about the lack of alignment between messages from leadership. But it does not allege that these ambiguities lead to the failure to generate, share, or store knowledge. I consider such examples nonetheless evidence of corporate epistemic vice. Employees at organizations that have the epistemic vice of fragmentation are likely to make such statements. Vice versa, employees at companies with the corresponding virtue are unlikely to make such statements. This is an approach familiar from psychology. Psychologists often measure latent traits such as personality traits by asking people about whether they agree with statements the endorsement of which would *reflect* the fact that they have the respective trait (DeVellis, 2016, 108 ff.). For instance, the survey item “I prefer meeting people to reading” may be used as part of an instrument to measure the personality trait of extroversion – even though endorsement of this item does not constitute extroversion, and could indeed be true of an introverted person who simply dislikes or is unable to read. Nonetheless, assessing latent traits through a number of such reflective items is a successful strategy in psychology and considered best practice (DeVellis, 2016, 108 ff.). I adopt this approach, by taking sentences reflective of corporate epistemic vice as evidence that the corporation is indeed epistemically vicious – even though the sentences do not contain explicit epistemic vice charges.

## Philosophical implications

The list of corporate vices emerging from this methodology is equally remarkable for what it contains as for what it does not contain. One observation is that in contrast to Baird and Calvards taxonomy, half of epistemic vices seem ‘faculty-like’ in that they are grounded in systems, processes, or structures rather than ‘trait-like’, i.e. grounded in traits of the organization that involve bad, or the absence of sufficiently good, epistemic motives: “Unreliability” is about the lack of reliable mechanisms for creating, sharing, and storing knowledge “Fragmentation” about challenges to information flows created by bureaucratic, matrixed, and hierarchical governance structures. “Complexity” concerns complex or otherwise inefficient processes undermining the creation, sharing, and storing of knowledge. The ‘faculty-like’ description of the factors are ultimately due to my interpretation. But the emphasis on faculties is well supported by the way employees discuss epistemic failings of their organizations in the respective reviews. The majority of reviews pertaining to these factors focus on how systems, processes, and structures lead to epistemic failings.

Of the six vices identified, two have close correlates in the existing literature. First “Incompetence”, which de Bruin (2015) uses to refer to epistemic vice generally, and which is used here to group comments related to the lack of knowledge of people to do their job well. Second, “Dishonesty”, which is closely related to the notion of epistemic malevolence used by Baird and Calvard (2019); which Baehr (2010) uses to denote an epistemic stance that is deeply opposed to the epistemic good of others; and which Meyer and Choo (Meyer and Choo, 2023) argue is best understood empirically in organizations as a particular type of deception.

The other four vices proposed by the present analysis have not been identified at all or only in aspects. While these vices have to my knowledge not been discussed as collective epistemic vices, the closely related ideas have been studied extensively in other disciplines. “Unreliability” refers to a lack of reliable systems for creating, sharing, and storing information. Day et al. (2009) provide an information systems perspective on impediments to information flow in the aftermath of Hurricane Katrina in the USA in 2005. They find that unreliability of information systems was a major impediment to the relief efforts. “Fragmentation” refers to organizational structures or interdepartmental dynamics that undermine effective knowledge sharing. The distinguishing feature of this vice is that it resides in the cultural environment and governance structures of the organization. Compare Essén, Knudsen, and Alvesson (2021) and Alvesson and Spicer (2012) for an organizational science perspective. “Ambiguity” relates to role conflict and ambiguity that undermine the creation and sharing of organizational knowledge. Role conflict and ambiguity are long-standing topics in the research of organizational behavior. In their seminal study, House and Rizzo (1972) showed that role ambiguity and role conflict negatively affect organizational effectiveness. “Complexity” concerns complex or otherwise inefficient processes that undermine the creation, sharing, or storing of organizational knowledge. Dekker (2016) argues that complexity of systems is a major cause of disasters. These four epistemic vices are ripe for detailed philosophical inquiry.

It is equally notable that several vices that have been discussed in the literature on collective epistemic vice are missing from the proposed taxonomy or framed substantially differently. For instance, from Baird and Calvard’s taxonomy (2019), neither epistemic insouciance, nor epistemic injustice or epistemic hubris emerged in the proposed taxonomy. There are some conceptual relationships, however. Epistemic insouciance is a lack of concern for epistemic goods. Most of the vices distinguished in the proposed terminology can be seen as an expression of such a lack of concern, e.g., hiring incompetent managers (“Incompetence”) or tolerating unreliable systems (“Unreliability”). Epistemic injustice may plausibly be exacerbated through hierarchy and bureaucracy and hence by “Fragmentation”. Epistemic hubris appears to be related to some dimensions of “Incompetence”, whose corresponding word cluster includes “arrogant”, “oblivious”, and “disrespectful”.^4^ Nonetheless, it is noteworthy that the extension of these three vices discussed by Baird and Calvard are substantially different from any of the vices emerging from the present analysis.

One explanation is that these vices are not salient features of corporations after all. Baird and Calvard’s list of vices draws on concepts that have been discussed in the philosophical literature on individual vice. Hence it would not be surprising to find that in collectives other vices are in fact salient. Yet we should not jump to the conclusion that epistemic insouciance and epistemic injustice are of little relevance at the collective level. Another option is that these vices are ‘stealthy’, in the sense people working in organizations that display these vices are blind to them (Cassam, 2015). Yet another option is that while these vices may not be frequent enough to register in the present analysis, they are nonetheless salient because organizations that suffer from them are prone to particularly destructive behavior.

There is evidence of all three mechanisms introduced above that may lead to epistemic vice. There is no clean mapping from the mechanisms to the vices, for the comments relating to each of the vices tend to be heterogeneous in this respect. However, there are tendencies. “Incompetence”, “Ambiguity”, and “Dishonesty” are typically attributed to groups of individuals in an organization, and often to leaders or managers. Hence these vices may often be realized in organizations in an ‘additive’ fashion. By contrast, comments attributing the vices of “Unreliability”, “Fragmentation” and “Complexity” tend to identify mechanisms, systems, processes, or governance structures as the locus of the vice. Hence these vices appear to be linked to the ‘interactionist’ model. At the same time, the latter two of these vices, “Fragmentation” and “Complexity” can only be attributed to collectives, not to individuals, fitting the ‘emergent’ model. The fact that these vices can only be attributed to collectives makes them ‘genuine’ collective vices. In the case of “Fragmentation”, the reason it can be considered an ‘emergent’ vice is that it resides in the organizational structure or interdepartmental dynamics of a corporation. “Complexity” refers to processes or information systems undermining the sharing of knowledge, which require a collective of others to share information with.

Incidentally, the analysis also provides a data point in the debate whether epistemic and moral vices are distinct. From the perspective of use in ordinary language, we can see clear overlaps in the case of some epistemic vices. For instance, “unethical” is among one of the five words closest in meaning to “Dishonesty”. “Incompetence” appears also to be a partly moralized notion, since it includes the terms “arrogant”, “disrespectful”, “spineless”, and “lazy”. By contrast, in the word clouds of the four other epistemic vices contain no obvious examples of moral language. This suggests that regardless of the questions whether there are systematic conceptual differences between moral and epistemic vices, employees treat epistemic and moral terms as interchangeable concerning some epistemic vices, while the use of others appears to be purely epistemic.

## Conclusion

This paper makes two contributions: To develop a methodology for determining which collective epistemic vices to posit, and to apply this methodology to corporations, resulting in a new taxonomy of corporate epistemic vice. In closing, I want to acknowledge some limitations of my analysis which point to opportunities for future research. The analysis is based on data about a particular type of collective, namely large corporations operating in the US. In other types of collectives, different epistemic vices may well be present. Future research may investigate corporations operating in other geographies, organizations of different types such as NGOs or government agencies, and organizations of different sizes, such as SMEs and startups.

Another limitation is that this research identifies epistemic vices by looking for patterns in trait word usage between companies. This methodology may fail to identify epistemic vices that would emerge from analyzing the views of different people within the same organization. For instance, the degree to which employees are subject to epistemic injustice varies within organizations, for instance based on markers such as gender or ethnicity. Hence it is perhaps no surprise that the methodology fails to pick up on the vice of epistemic injustice. Future research should take the perspectives of different groups within organizations into account.

Yet another limitation is that the analysis is based on the perspective of employees and former employees of organizations. Cassam (2015) has argued that some epistemic vices may be ‘stealthy’ in the sense that in virtue of being subject to the vice, their bearers may be oblivious to them. One way in which organizations can display epistemic vice is because some of their members have the epistemic vice. To the extent that epistemic vices are stealthy, and respondents suffer from the respective vices themselves, the methodology would fail to detect them. Future research may draw on data by external stakeholders of organizations to detect stealthy vices.

## Electronic Supplementary Material

Below is the link to the electronic supplementary material.

Supplementary Material 1

## Data Availability

https://osf.io/e98kb/?view_only=705ca360bc124d2baee9f062228507c0

## Footnotes

1 I am grateful to an anonymous reviewer for prompting me to clarify this point.

2 I am grateful to an anonymous reviewer for pushing me on this point.

3 I am grateful for an anonymous reviewer for pushing me to clarify this point.

4 I am grateful to an anonymous reviewer for pointing these conceptual links out to me.
