# Supplementary material for: The epistemic vices of corporations
Source: Synthese. 2023 Apr 20;201(5):148. doi: 10.1007/s11229-023-04133-2 (PMC10117242; doi:10.1007/s11229-023-04133-2)
Supplement: Supplementary file 1 — Supplementary Material 1 [file 11229_2023_4133_MOESM1_ESM.docx]

SUPPLEMENTAL INFORMATION

THE EPISTEMIC VICES OF CORPORATIONS

This supplementary information file provides additional information, including on methods and results to support the findings presented in the main text of the article. The article proposes an empirical approach to identifying epistemic vices in corporations, by analyzing a large dataset of online employee reviews. The article aims to surface epistemic vices that are attributed to corporations by its own members and reduce the number of vices to the minimum required to describe differences between corporations.

*Table of contents*

[1. Data](#_472ffflewvj3) 2

[2. Section 4.1: Extract adjectives from employee review dataset](#_3l3mc1ldon6g) 2

[3. Section 4.1: Assess the interrater-reliability for the labeling of adjectives as epistemic](#_opgn37h8c9na) 2

[4. Section 4.1: Identifying word clusters related to epistemic vice](#_38eqomqm3rnt) 5

[5. Section 4.2: Measuring ascriptions of trait word clusters to companies](#_egad2ypakjgw) 11

[6. Section 4.2: Conduct factor analysis of wordcount data](#_sanrzpb2wkv8) 12

[7. Section 4.2: Extracting example sentences](#_keb2bry4pq75) 21

## Data

The data used are 1,36 million reviews from the online review platform Glassdoor, collected about US companies between 2008 and 2020, which are available from the company upon request (contact the author or Glassdoor directly). The author obtained access to the data free of charge for academic purposes.

All code and intermediate data and outputs are available on OSF: <https://osf.io/e98kb/?view_only=705ca360bc124d2baee9f062228507c0>

## Section 4.1: Extract adjectives from employee review dataset

*Text Cleaning:* I first extract only relevant strings from the employee review dataset to speed up analysis. I use a regular expression pattern to identify only alphanumeric and hyphenated text with three or more characters.

*Adjective Extraction*: Adjective extraction is achieved with the python library *spaCy*, an industrial-strength natural language processing library, to identify all words in the text that are classified as adjectives. The output is a list of adjectives, with each word in the list converted to lowercase and represented by its lemma. There are 23.500 strings classified as adjectives, which occur 3.966.075 times. The large number of adjectives is due to the fact that many adjectives are misspelled, and misspelled in many different ways, yet *spaCey* is still able to identify the strings as adjectives. To keep the manual classification task manageable, I focus only on adjectives that occur at least once in every 10.000 reviews. This results in a list of 960 adjectives. The full list of adjectives is included in the OSF repository.

## Section 4.1: Assess the interrater-reliability for the labeling of adjectives as epistemic

I assess interrater-reliability for the labeling of adjectives as epistemic. Three researchers rated the adjectives commonly used in the dataset independently, based on whether they characterized the way an organization or people in an organization deal with information, and if they have a negative valence. The goal is to determine whether the ratings cohere sufficiently and to identify adjectives for further analysis. Fleiss' Kappa to determine interrater-reliability, which was found to be 0,71, which meets the customary threshold of > 0,6.

Where judgments diverged, I consolidated ratings by simple majority and created a list of adjectives that were selected by at least 2 of the 3 researchers. This methodology led me to select 82 adjectives, or 9% of the adjectives reviewed, for further analysis. Examples include “stupid”, “disorganized”, and “rigid”. The table S1 shows the list of selected adjectives. The OSF repository contains the file with the individual ratings by all three researchers.

Table S1: List of adjectives with a negative valence classified as relating to epistemic issues. Counts are the number of times the lemma of the adjective occurs in the dataset (1,36 million reviews)

| Adjective | Count |
| --- | --- |
| political | 13874 |
| unrealistic | 12282 |
| unprofessional | 7897 |
| inconsistent | 7300 |
| incompetent | 6710 |
| lazy | 6472 |
| disorganized | 6387 |
| unorganized | 6311 |
| bureaucratic | 5471 |
| inexperienced | 3429 |
| clueless | 3239 |
| stupid | 2771 |
| complex | 2683 |
| chaotic | 2440 |
| inefficient | 2238 |
| confusing | 2185 |
| fake | 2108 |
| insane | 2082 |
| rigid | 1991 |
| antiquated | 1976 |
| complicated | 1950 |
| unqualified | 1694 |
| ineffective | 1602 |
| hierarchical | 1580 |
| disconnected | 1520 |
| arrogant | 1463 |
| condescending | 1350 |
| dysfunctional | 1315 |
| dishonest | 1313 |
| blind | 1287 |
| dumb | 1239 |
| siloed | 1177 |
| inept | 1166 |
| unreliable | 1096 |
| biased | 1055 |
| pointless | 1022 |
| vague | 1019 |
| arbitrary | 986 |
| cliquey | 941 |
| overwhelmed | 909 |
| subjective | 894 |
| confused | 858 |
| ignorant | 843 |
| isolated | 656 |
| complacent | 654 |
| irrelevant | 608 |
| incorrect | 604 |
| secret | 595 |
| disjointed | 591 |
| unaware | 580 |
| secretive | 578 |
| cliquish | 542 |
| convoluted | 540 |
| discouraging | 536 |
| obsolete | 533 |
| uneven | 530 |
| incapable | 523 |
| ambiguous | 518 |
| conflicting | 516 |
| misleading | 504 |
| reactionary | 433 |
| fragmented | 418 |
| gossipy | 413 |
| manipulative | 412 |
| sloppy | 397 |
| oblivious | 392 |
| discriminatory | 392 |
| unskilled | 374 |
| hypocritical | 368 |
| skewed | 367 |
| inaccurate | 366 |
| matrixed | 362 |
| confidential | 362 |
| unresponsive | 361 |
| oppressive | 359 |
| paranoid | 353 |
| contradictory | 349 |
| decentralized | 346 |
| deceptive | 332 |
| untrustworthy | 331 |
| tribal | 328 |
| unstructured | 325 |

## Section 4.1: Identifying word clusters related to epistemic vice

*Training a Word2Vec Model:* Word2Vec is a popular language modeling technique used in natural language processing and computer vision. It is a type of neural network that is trained to predict contextually similar words for a given word in a large corpus of text. The model represents words as vectors in a high-dimensional space, where semantically similar words are positioned close to each other. This allows the model to capture the relationships between words and their usage in context. The resulting word embeddings can then be used for a variety of NLP tasks, such as text classification, machine translation, and named entity recognition. Word2Vec has shown to be effective in capturing semantic relationships between words and has been widely used in various NLP applications. The purpose of this step is to find semantically similar adjectives to the adjectives selected as epistemic-vice-related. To ensure that the model is robust, I merge both pros and cons reviews into a single set of reviews, preprocess the reviews, and create the model.

I use the *Gensim* package, a popular python library for natural language tasks, to train a Word2Vec model based on the employee review data. The training process involves learning the relationships between words in the corpus and representing them as vectors in a high-dimensional space. To start the training process, I need to specify the window size, which determines the number of surrounding words to consider for each target word. Additionally, I need to specify the minimum count of words to be included in the model, to ensure that rare words are not overrepresented. Finally, I need to specify the training algorithm, either CBOW (Continuous Bag-of-Words) or skip-gram. The main difference between CBOW and skip-gram is the direction of the prediction task. In CBOW, the goal is to predict the target word given the surrounding context words, whereas in skip-gram, the goal is to predict the surrounding context words given the target word. CBOW is faster and more efficient than skip-gram, as it predicts one target word given many context words at once. However, skip-gram has been shown to perform better in capturing rare words and semantic relationships between words. In general, the choice between CBOW and skip-gram depends on the specific NLP task and the size of the corpus. CBOW is recommended for larger corpora, while skip-gram is recommended for smaller corpora or when rare words need to be captured effectively. While our corpus is large, I try to capture comparatively rare words, and therefore choose a skip-gram architecture. I choose the customary window size of 5. The model is trained on the preprocessed reviews using a minimum count of 30 to exclude words that appear too few times. I train the model for five epochs, allowing it to learn the relationships between words in the corpus over multiple iterations. The trained model will be used in the next step to identify semantically similar adjectives. The saved model is included in the OSF repository.

I use the Word2Vec model to identify word clusters that are semantically related to the adjectives selected in the previous step. The aim is to identify clusters of words that are semantically similar to the selected adjectives and that could indicate epistemic vice in organizations.

I loop over each of the 82 adjectives selected in the previous step, and use the Word2Vec model to find the five words that are most similar in meaning. The result is a dictionary with 82 keys, each representing an adjective, and the values are lists of the five most similar words. The implementation details are contained in the python notebook contained in the OSF repository.

To count how often each trait word cluster occurs in the dataset, the first step involves using the package *Scikit-learn* (sklearn) to count the number of times words from each word cluster appear in each review. sklearn is a popular open-source machine learning library for Python that provides a range of algorithms and tools for various machine learning tasks. One of the useful tools provided by sklearn is the CountVectorizer model. CountVectorizer is a text feature extraction tool that transforms a collection of text documents into a numerical feature matrix, where each row represents a document and each column represents a feature (word) in the vocabulary. The values in the matrix represent the number of times a word appears in each document.

I convert the word clusters into a dictionary for use in the CountVectorizer model. The vocabulary is made up of all the values in the dictionary, and the CountVectorizer is trained on the vocabulary to count the number of words in the vocabulary within the review dataset. The resulting count columns are transformed into a dataframe, merged with the rest of the data, and new variables are created to sum up the counts of words belonging to each word cluster. Finally, the proportion of reviews that mention at least one word from a relevant word cluster is calculated, and this is found to be 16%. The final step involves converting the cluster count into a dataframe and adding a column with all the relevant words for each cluster. The list of words is then converted into a single string and the table is sorted by word cluster frequency. Table S2 contains the list of word clusters, how often they occur, and the trait words that make up each cluster (selected adjective + five words identified through the Word2Vec model)

Table S2: Word clusters consisting of adjectives and additional words similar in meaning based on a Word2Vec model of the dataset.

​​

| Word cluster | Count | All trait words |
| --- | --- | --- |
| political | 18828 | politicized, politcal, beauracratic, beaucratic, bureaucratic, political |
| unrealistic | 17184 | unreasonable, unreachable, unattainable, unachievable, unobtainable, unrealistic |
| unorganized | 16374 | disorganized, unorganised, disorganised, messy, chaotic, unorganized |
| ineffective | 15521 | inept, ineffectual, inefficient, weak, incompetent, ineffective |
| unstructured | 15370 | disorganized, chaotic, unorganized, unfocused, uncoordinated, unstructured |
| chaotic | 15369 | disorganized, frenetic, unfocused, unstructured, unorganized, chaotic |
| disorganized | 15266 | unorganized, chaotic, unstructured, disorganised, uncoordinated, disorganized |
| unprofessional | 14762 | disrespectful, condescending, immature, racist, childish, unprofessional |
| secretive | 14275 | hush, disengaging, political, uncommunicative, distrusting, secretive |
| inefficient | 14145 | ineffective, antiquated, outdated, cumbersome, archaic, inefficient |
| lazy | 13173 | unintelligent, unmotivated, incompetent, nosey, bossy, lazy |
| confusing | 12939 | convoluted, complicated, frustrating, cumbersome, jumbled, confusing |
| unqualified | 12632 | underqualified, inexperienced, incompetent, untrained, uneducated, unqualified |
| inexperienced | 12037 | unexperienced, unqualified, untrained, underqualified, incompetent, inexperienced |
| ignorant | 11809 | uneducated, unintelligent, arrogant, incompetent, disrespectful, ignorant |
| antiquated | 11806 | outdated, archaic, ancient, primitive, dated, antiquated |
| clueless | 11627 | oblivious, incompetent, inept, unaware, uninvolved, clueless |
| insane | 10746 | ridiculous, absurd, outrageous, ungodly, unreal, insane |
| incompetent | 10149 | inept, unqualified, uneducated, spineless, incompetant, incompetent |
| discouraging | 9979 | disheartening, frustrating, demotivating, disappointing, demoralizing, discouraging |
| incapable | 9816 | inept, incompetent, unsuited, capable, unaware, incapable |
| obsolete | 9618 | outdated, ancient, antiquated, deprecated, primitive, obsolete |
| dysfunctional | 9494 | disfunctional, toxic, disjointed, ineffective, fractured, dysfunctional |
| inconsistent | 9444 | inconsistant, inconstant, erratic, sporadic, inconsistency, inconsistent |
| hierarchical | 9417 | hierarchal, bureaucratic, hierarchial, hierarchy, heirarchy, hierarchical |
| inept | 9366 | incompetent, ineffective, spineless, ineffectual, impotent, inept |
| complicated | 9143 | complex, convoluted, cumbersome, confusing, unintuitive, complicated |
| matrixed | 9064 | matrix, layered, hierarchical, bureaucratic, decentralized, matrixed |
| tribal | 8949 | institutional, knowledge, hoarding, hording, brainpower, tribal |
| pointless | 8912 | useless, meaningless, unnecessary, redundant, silly, pointless |
| sloppy | 8701 | disorganized, haphazard, amateurish, shoddy, messy, sloppy |
| convoluted | 8572 | cumbersome, complicated, confusing, byzantine, inefficient, convoluted |
| unreliable | 8172 | undependable, flakey, faulty, unorganized, sporadic, unreliable |
| unaware | 7703 | oblivious, clueless, incapable, disconnected, detached, unaware |
| biased | 7370 | unfair, prejudiced, prejudicial, discriminatory, racist, biased |
| bureaucratic | 7263 | bureacratic, beurocratic, beaurocratic, beauracratic, hierarchical, bureaucratic |
| unskilled | 6628 | inexperienced, untrained, uneducated, unqualified, underqualified, unskilled |
| complex | 6545 | complicated, intricate, cumbersome, matrixed, unwieldy, complex |
| conflicting | 6262 | contradictory, contradicting, differing, unclear, directions, conflicting |
| overwhelmed | 6228 | overloaded, frustrated, overburdened, swamped, stressed, overwhelmed |
| dishonest | 5531 | deceitful, untrustworthy, unethical, deceptive, unscrupulous, dishonest |
| irrelevant | 5151 | meaningless, useless, unrelated, pointless, unimportant, irrelevant |
| condescending | 5034 | disrespectful, demeaning, belittling, abrasive, cruel, condescending |
| dumb | 4849 | stupid, idiotic, silly, pettiest, lame, dumb |
| ambiguous | 4843 | vague, unclear, undefined, contradictory, unorthodox, ambiguous |
| vague | 4800 | contradictory, ambiguous, unclear, definitions, nebulous, vague |
| fragmented | 4640 | siloed, fractured, disjointed, segmented, silo, fragmented |
| stupid | 4593 | dumb, idiotic, silly, asinine, stupidest, stupid |
| deceptive | 4510 | dishonest, deceitful, deceiving, misleading, shady, deceptive |
| cliquish | 4443 | cliquey, clicky, clickish, clique, gossipy, cliquish |
| oblivious | 4200 | unaware, clueless, blissfully, ineffectual, disassociated, oblivious |
| disconnected | 4180 | detached, distanced, disassociated, distant, disengaged, disconnected |
| rigid | 4081 | ridged, restrictive, regimented, inflexible, stringent, rigid |
| siloed | 4067 | silo, segmented, fragmented, silod, fractured, siloed |
| complacent | 3949 | jaded, stagnant, unmotivated, cynical, entrenched, complacent |
| confused | 3786 | disillusioned, unsure, bewildered, frustrated, dazed, confused |
| gossipy | 3630 | catty, cliquey, caddy, clicky, childish, gossipy |
| decentralized | 3539 | matrixed, siloed, heirarchical, fragmented, centralized, decentralized |
| cliquey | 3470 | clicky, cliquish, gossipy, caddy, catty, cliquey |
| contradictory | 3463 | contradicting, conflicting, vague, ambiguous, contradict, contradictory |
| disjointed | 3067 | fragmented, fractured, siloed, segmented, disfunctional, disjointed |
| blind | 2892 | atrocities, willfully, cowardly, eye, deaf, blind |
| subjective | 2857 | opaque, nebulous, flawed, arbitrary, laddering, subjective |
| hypocritical | 2816 | disingenuous, judgmental, deceitful, condescending, degrading, hypocritical |
| misleading | 2576 | deceiving, deceptive, mislead, misrepresented, vague, misleading |
| untrustworthy | 2554 | dishonest, conniving, vindictive, manipulative, deceitful, untrustworthy |
| manipulative | 2554 | vindictive, conniving, dishonest, untrustworthy, deceitful, manipulative |
| discriminatory | 2518 | discriminating, racist, prejudicial, prejudiced, sexist, discriminatory |
| arbitrary | 2456 | arbitrarily, illogical, subjective, opaque, massaged, arbitrary |
| fake | 2321 | phony, fakes, phoney, inauthentic, fabricates, fake |
| reactionary | 2284 | reactive, shortsighted, reactively, reacting, tactical, reactionary |
| inaccurate | 2173 | incorrect, accurate, factually, incomplete, exaggerated, inaccurate |
| arrogant | 2073 | smug, pompous, egotistical, narcissistic, cocky, arrogant |
| isolated | 1847 | secluded, segregated, isolating, isolation, separated, isolated |
| incorrect | 1549 | inaccurate, erroneous, incomplete, incorrectly, grammatical, incorrect |
| unresponsive | 1520 | unhelpful, uncommunicative, inaccessible, unapproachable, indifferent, unresponsive |
| uneven | 1440 | unbalanced, imbalanced, unevenly, unequal, inequitable, uneven |
| paranoid | 1331 | insecure, frightened, fearful, cowardly, distrustful, paranoid |
| skewed | 1196 | slanted, lopsided, weighted, tilted, unbalanced, skewed |
| secret | 895 | victorias, henri, victoria, bendel, bw, secret |
| confidential | 865 | anonymously, witnesses, anonymous, confidentially, discreet, confidential |
| oppressive | 836 | authoritarian, militaristic, militant, suffocating, caustic, oppressive |

## Section 4.2: Measuring ascriptions of trait word clusters to companies

Next, the counts for each cluster are aggregated at the company level. I calculate the mean of each cluster column for each unique employer name and store the result in a dataframe, which is available as a CSV file in the OSF repository.

After aggregating the cluster counts, I analyze the average correlations between the cluster counts. The average correlation is 0,06. Below is a histogram of the proportion of reviews containing any cluster term per company to visualize the frequency of companies encountering epistemic vice terms.

Figure S1: Histogram of the proportion of reviews containing any cluster term per company to visualize the frequency of companies encountering epistemic vice terms.


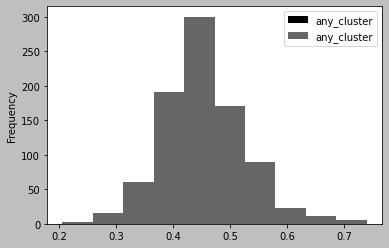


## Section 4.2: Conduct factor analysis of wordcount data

In this section, the word clusters from customer reviews are grouped based on a factor analysis. The objective of this analysis is to identify groups of word clusters that tend to co-vary across companies. If the frequency of one word cluster is high, the frequency of other word clusters in the same group is also likely to be high.

I use the python package *Factor Analyzer*, a Python library for factor analysis. Factor analysis is a statistical method for exploring the underlying structure of a set of variables. Factor analysis can be used to identify the underlying factors or latent variables that are responsible for the covariance structure in a set of observed variables. I use factor analysis to identify the latent structure in a set of variables, for example, in a survey data where the responses to various questions are the observed variables, and the underlying factors are the latent variables that explain the relationships among the questions.

First, we need to determine the number of factors to extract. I use a scree plot, a graphical representation of the eigenvalues of the factors in a factor analysis. The eigenvalues represent the amount of variance in the data that is explained by each factor, and the Scree plot displays these values in a bar graph. To determine the number of factors to extract, I look for the "elbow" in the plot, which is the point where the eigenvalues start to level off, at a point where Eigenvalues > 1, as those factors explain more variance in the data than the error. The scree plot suggests the extraction of six factors. The scree plot suggests the extraction of six factors (Figure S2). It is important to note that the interpretation of the scree plot is subjective and can vary depending on the researcher's objectives and the nature of the data. Thus, I use the scree plot in conjunction with a check for plausibility based on the interpretability of the factors extracted in the next step to make an informed decision on the number of factors to extract, which further supports the decision to extract six factors.

Figure S2: First scree plot based on 82 word clusters, suggesting six factors.

**
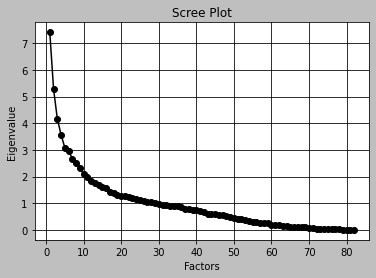
**

The factor analysis process is iterative, and the goal is to find meaningful clusters that co-vary substantially and don't co-vary with word clusters assigned to other factors. To achieve this, word clusters were eliminated from the analysis based on established criteria used in psychometrics.

The final result after four rounds of factor analysis is a list of 31 word clusters grouped into six factors. These factor mappings form the basis for the description of corporate epistemic vices in the next section.

The data showed a small range of correlations, with the smallest correlation being -0,17 and the average of average correlations being 0,05. This indicates that the word clusters tend to have weak or limited relationships with each other.

**First factor analysis**

The next step in this analysis is to assess the suitability of the data for factor analysis. This is done by calculating the Kaiser-Meyer-Olkin (KMO) measure. The KMO measures the proportion of variance among all the variables that is explained by the intercorrelations. A KMO value greater than 0,6 indicates that the data is suitable for factor analysis. In this analysis, the KMO value was 0,68, which falls in the acceptable range, indicating that the data is suitable for factor analysis.

After assessing the suitability of the data, a factor analysis was conducted using oblique rotation, with the aim of extracting six factors (Table S3). The factor analysis was able to explain 29% of the variance in the data, as indicated by the factor variance for each factor.

Table S3: Factor loadings of first factor analysis based on 82 word clusters.

| Word cluster | FA1 | FA2 | FA3 | FA4 | FA5 | FA6 |
| --- | --- | --- | --- | --- | --- | --- |
| political_cluster | 0,09 | -0,11 | 0,71 | 0,02 | 0,03 | 0,03 |
| unrealistic_cluster | 0,08 | 0,09 | -0,04 | 0,08 | 0,04 | 0,07 |
| unprofessional_cluster | 0,04 | 0,15 | -0,18 | 0,10 | -0,13 | 0,24 |
| inconsistent_cluster | -0,06 | 0,06 | -0,03 | 0,06 | -0,03 | 0,07 |
| incompetent_cluster | 0,92 | 0,00 | -0,01 | -0,09 | 0,01 | -0,01 |
| lazy_cluster | 0,70 | -0,05 | -0,09 | -0,07 | -0,02 | 0,01 |
| disorganized_cluster | 0,00 | 0,99 | 0,00 | 0,00 | -0,01 | 0,01 |
| unorganized_cluster | -0,02 | 0,96 | -0,01 | 0,00 | -0,01 | 0,00 |
| bureaucratic_cluster | -0,04 | -0,04 | 0,74 | -0,03 | -0,10 | 0,06 |
| inexperienced_cluster | 0,86 | 0,03 | 0,02 | -0,10 | 0,01 | -0,03 |
| clueless_cluster | 0,82 | -0,01 | 0,02 | 0,17 | 0,02 | -0,03 |
| stupid_cluster | 0,11 | 0,04 | -0,04 | 0,11 | -0,04 | 0,10 |
| complex_cluster | 0,00 | -0,01 | 0,12 | 0,49 | 0,05 | -0,17 |
| chaotic_cluster | 0,00 | 0,99 | 0,00 | 0,00 | 0,00 | 0,00 |
| inefficient_cluster | 0,03 | 0,03 | -0,01 | 0,64 | -0,12 | 0,15 |
| confusing_cluster | 0,00 | -0,05 | -0,01 | 0,58 | 0,11 | -0,08 |
| fake_cluster | 0,05 | -0,03 | -0,06 | 0,05 | -0,02 | 0,08 |
| insane_cluster | 0,00 | 0,06 | -0,12 | 0,15 | 0,00 | 0,07 |
| rigid_cluster | 0,04 | -0,02 | 0,04 | 0,02 | 0,01 | 0,02 |
| antiquated_cluster | -0,02 | -0,03 | -0,06 | 0,52 | -0,17 | 0,16 |
| complicated_cluster | -0,04 | -0,03 | 0,03 | 0,65 | 0,07 | -0,13 |
| unqualified_cluster | 0,87 | 0,04 | 0,02 | -0,12 | 0,00 | -0,02 |
| ineffective_cluster | 0,71 | 0,01 | 0,10 | 0,17 | 0,05 | 0,00 |
| hierarchical_cluster | -0,05 | -0,04 | 0,69 | -0,03 | -0,12 | 0,06 |
| disconnected_cluster | 0,02 | 0,08 | 0,00 | 0,30 | 0,06 | -0,04 |
| arrogant_cluster | 0,08 | -0,09 | 0,05 | 0,10 | -0,01 | 0,15 |
| condescending_cluster | 0,05 | 0,06 | -0,17 | 0,11 | -0,12 | 0,19 |
| dysfunctional_cluster | 0,01 | 0,17 | 0,10 | 0,11 | 0,05 | 0,02 |
| dishonest_cluster | 0,02 | -0,01 | -0,01 | 0,06 | 0,07 | 0,54 |
| blind_cluster | 0,08 | 0,02 | 0,03 | 0,08 | -0,03 | 0,09 |
| dumb_cluster | 0,12 | 0,04 | 0,00 | 0,09 | -0,04 | 0,11 |
| siloed_cluster | 0,02 | 0,14 | 0,45 | 0,09 | 0,20 | -0,07 |
| inept_cluster | 0,88 | 0,00 | 0,01 | 0,07 | 0,02 | 0,00 |
| unreliable_cluster | 0,02 | 0,55 | -0,04 | -0,01 | -0,05 | 0,00 |
| biased_cluster | 0,00 | 0,02 | 0,00 | 0,06 | -0,05 | 0,16 |
| pointless_cluster | 0,15 | 0,02 | -0,01 | 0,24 | -0,06 | -0,05 |
| vague_cluster | 0,00 | -0,03 | -0,02 | -0,02 | 0,88 | 0,03 |
| arbitrary_cluster | 0,05 | -0,02 | 0,02 | 0,18 | 0,08 | 0,04 |
| cliquey_cluster | -0,04 | 0,13 | 0,00 | 0,02 | -0,14 | 0,23 |
| overwhelmed_cluster | -0,01 | 0,10 | 0,01 | 0,10 | -0,03 | 0,04 |
| subjective_cluster | 0,08 | 0,00 | 0,00 | 0,17 | 0,10 | 0,07 |
| confused_cluster | 0,04 | 0,10 | 0,06 | 0,11 | 0,03 | 0,07 |
| ignorant_cluster | 0,76 | -0,03 | -0,07 | 0,02 | -0,07 | 0,12 |
| isolated_cluster | 0,02 | -0,07 | 0,04 | 0,06 | 0,11 | 0,03 |
| complacent_cluster | 0,12 | -0,05 | 0,09 | 0,13 | -0,05 | 0,07 |
| irrelevant_cluster | 0,15 | 0,00 | -0,03 | 0,19 | -0,11 | -0,01 |
| incorrect_cluster | 0,11 | 0,08 | -0,03 | 0,07 | 0,08 | 0,00 |
| secret_cluster | 0,00 | -0,04 | 0,07 | 0,01 | 0,00 | 0,01 |
| disjointed_cluster | -0,02 | 0,13 | 0,42 | 0,08 | 0,20 | -0,09 |
| unaware_cluster | 0,16 | 0,10 | 0,05 | 0,41 | 0,04 | 0,00 |
| secretive_cluster | 0,13 | -0,12 | 0,48 | 0,05 | 0,10 | 0,02 |
| cliquish_cluster | 0,03 | 0,10 | 0,01 | 0,03 | -0,11 | 0,15 |
| convoluted_cluster | -0,01 | 0,01 | -0,01 | 0,67 | 0,07 | -0,05 |
| discouraging_cluster | 0,10 | 0,01 | 0,07 | 0,21 | 0,13 | -0,01 |
| obsolete_cluster | -0,02 | -0,05 | -0,04 | 0,48 | -0,15 | 0,18 |
| uneven_cluster | -0,03 | 0,01 | -0,03 | 0,11 | -0,05 | 0,04 |
| incapable_cluster | 0,85 | -0,02 | -0,01 | 0,06 | -0,03 | 0,00 |
| ambiguous_cluster | -0,01 | 0,00 | -0,03 | -0,01 | 0,88 | 0,03 |
| conflicting_cluster | 0,03 | 0,01 | 0,02 | 0,00 | 0,66 | 0,08 |
| misleading_cluster | -0,01 | -0,04 | -0,10 | 0,13 | 0,33 | 0,07 |
| reactionary_cluster | 0,05 | 0,00 | 0,05 | 0,05 | 0,07 | 0,07 |
| fragmented_cluster | 0,01 | 0,13 | 0,47 | 0,10 | 0,23 | -0,07 |
| gossipy_cluster | -0,05 | 0,16 | -0,04 | 0,02 | -0,15 | 0,23 |
| manipulative_cluster | 0,00 | 0,02 | 0,05 | -0,03 | 0,04 | 0,85 |
| sloppy_cluster | 0,00 | 0,57 | -0,01 | 0,04 | 0,02 | -0,02 |
| oblivious_cluster | 0,23 | 0,05 | 0,06 | 0,27 | 0,02 | -0,01 |
| discriminatory_cluster | 0,00 | 0,05 | 0,01 | -0,01 | -0,05 | 0,15 |
| unskilled_cluster | 0,34 | 0,10 | 0,05 | -0,11 | -0,01 | -0,01 |
| hypocritical_cluster | -0,07 | 0,09 | -0,12 | 0,15 | -0,05 | 0,20 |
| skewed_cluster | 0,02 | 0,02 | 0,02 | 0,02 | -0,01 | 0,06 |
| inaccurate_cluster | 0,08 | 0,05 | -0,05 | 0,12 | 0,07 | -0,03 |
| matrixed_cluster | -0,03 | 0,01 | 0,75 | -0,03 | -0,09 | 0,03 |
| confidential_cluster | 0,01 | 0,03 | -0,03 | 0,09 | -0,03 | -0,04 |
| unresponsive_cluster | 0,06 | 0,01 | 0,01 | 0,01 | -0,02 | -0,01 |
| oppressive_cluster | -0,02 | -0,04 | 0,02 | 0,06 | 0,00 | 0,11 |
| paranoid_cluster | 0,08 | 0,02 | 0,02 | 0,05 | -0,02 | 0,00 |
| contradictory_cluster | -0,04 | -0,04 | -0,04 | 0,05 | 0,52 | 0,10 |
| decentralized_cluster | 0,00 | 0,14 | 0,43 | 0,01 | 0,13 | -0,12 |
| deceptive_cluster | 0,00 | -0,05 | -0,09 | 0,06 | 0,10 | 0,56 |
| untrustworthy_cluster | 0,00 | 0,02 | 0,05 | -0,03 | 0,04 | 0,85 |
| tribal_cluster | 0,09 | -0,01 | 0,10 | 0,07 | 0,05 | 0,03 |
| unstructured_cluster | 0,01 | 0,99 | 0,00 | -0,01 | 0,00 | 0,00 |

The factor analysis revealed that some of the word clusters didn't show a strong association with any of the six factors. I eliminated clusters which loaded less than 0,4 on any factor, shown in Table S4.

Table S4: Clusters eliminated from analysis because of primary loadings < 0,4 in first factor analysis

| Word cluster | Primary loading |
| --- | --- |
| stupid_cluster | 0,11 |
| rigid_cluster | 0,04 |
| dumb_cluster | 0,12 |
| incorrect_cluster | 0,11 |
| unskilled_cluster | 0,34 |
| unresponsive_cluster | 0,06 |
| paranoid_cluster | 0,08 |
| unrealistic_cluster | 0,09 |
| dysfunctional_cluster | 0,17 |
| secret_cluster | 0,07 |
| tribal_cluster | 0,10 |
| insane_cluster | 0,15 |
| disconnected_cluster | 0,30 |
| pointless_cluster | 0,24 |
| arbitrary_cluster | 0,18 |
| overwhelmed_cluster | 0,10 |
| subjective_cluster | 0,17 |
| confused_cluster | 0,11 |
| complacent_cluster | 0,13 |
| irrelevant_cluster | 0,19 |
| discouraging_cluster | 0,21 |
| uneven_cluster | 0,11 |
| oblivious_cluster | 0,27 |
| inaccurate_cluster | 0,12 |
| confidential_cluster | 0,09 |
| isolated_cluster | 0,11 |
| misleading_cluster | 0,33 |
| reactionary_cluster | 0,07 |
| unprofessional_cluster | 0,24 |
| inconsistent_cluster | 0,07 |
| fake_cluster | 0,08 |
| arrogant_cluster | 0,15 |
| condescending_cluster | 0,19 |
| blind_cluster | 0,09 |
| biased_cluster | 0,16 |
| cliquey_cluster | 0,23 |
| cliquish_cluster | 0,15 |
| gossipy_cluster | 0,23 |
| discriminatory_cluster | 0,15 |
| hypocritical_cluster | 0,20 |
| skewed_cluster | 0,06 |
| oppressive_cluster | 0,11 |

In addition, I eliminated word clusters from the analysis with unacceptably high cross-loadings on other factors. The criterion was that the difference between the primary loading and the second-largest loading needs to be at least 0,3. This leads to the elimination of five word clusters, as shown in Table S5.

Table S5: Clusters eliminated from analysis in first factor analysis because difference between primary loadings and second largest loadings < 0,3

| Word cluster | Difference between primary loading and second highest loading |
| --- | --- |
| decentralized_cluster | 0,29 |
| siloed_cluster | 0,26 |
| unaware_cluster | 0,26 |
| fragmented_cluster | 0,24 |
| disjointed_cluster | 0,22 |

The result of the first factor analysis is a list of 35 factors.

**Second factor analysis**

The process described for the first factor analysis is repeated. The KMO for the second factor analysis was 0,76, well in the acceptable range. The six factors explain 62% of the variance in the remaining 35 word clusters. One cluster was eliminated because its primary loading was < 0,4: obsolete_cluster (0,37). Secretive_cluster and antiquated_cluster were eliminated because of the cross loading criterion. 32 factors remain.

**Third factor analysis**

The process described for the first factor analysis is repeated a second time. The KMO for the third factor analysis was 0,78, well in the acceptable range. The six factors explain 67% of the variance in the remaining 32 word clusters. inefficient_cluster was eliminated because of the cross loading criterion. 31 factors remain.

**Fourth factor analysis**

The process described for the first factor analysis is repeated a second time. The KMO for the fourth factor analysis was 0,79, well in the acceptable range. I repeated the dimensionality analysis: Figure S3 shows the scree plot for the remaining 31 factors. The plot clearly suggests the extraction of 6 factors.

Figure S3: Final scree plot based on 31 word clusters, suggesting 6 factors.


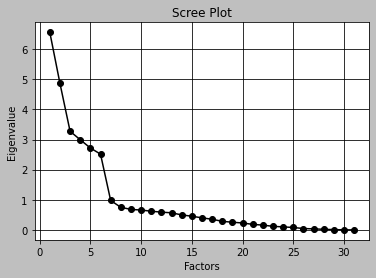


The six factors explain 69% of the variance in the remaining 31 word clusters. All clusters pass the inclusion criteria. Therefore, as in the previous analysis, 31 factors remain in our final analysis. The loadings of the final factor analysis are presented in Table S5 (also in the main paper, Table 1).

Table S5: Results of the final factor analysis extracting six factors from counts per company of 31 word clusters, using an oblimin rotation.

| Word cluster | Incompetence | Unreliability | Fragmentation | Ambiguity | Complexity | Dishonesty |
| --- | --- | --- | --- | --- | --- | --- |
| incompetent_cluster | 0,92 | 0,00 | -0,02 | -0,01 | -0,05 | 0,01 |
| inept_cluster | 0,91 | 0,00 | 0,01 | 0,02 | 0,04 | -0,01 |
| incapable_cluster | 0,87 | -0,01 | 0,01 | -0,03 | 0,04 | 0,01 |
| unqualified_cluster | 0,83 | 0,03 | 0,00 | -0,01 | -0,06 | -0,01 |
| clueless_cluster | 0,83 | -0,01 | 0,01 | 0,02 | 0,06 | -0,02 |
| inexperienced_cluster | 0,83 | 0,02 | 0,01 | 0,00 | -0,05 | -0,03 |
| ignorant_cluster | 0,76 | -0,03 | -0,05 | -0,05 | -0,02 | 0,07 |
| ineffective_cluster | 0,74 | 0,01 | 0,07 | 0,06 | 0,11 | 0,00 |
| lazy_cluster | 0,71 | -0,03 | -0,05 | 0,00 | -0,07 | -0,01 |
| unstructured_cluster | 0,01 | 1,00 | 0,00 | 0,01 | -0,01 | 0,00 |
| disorganized_cluster | 0,00 | 1,00 | 0,00 | -0,01 | 0,00 | 0,00 |
| chaotic_cluster | 0,00 | 1,00 | 0,00 | 0,01 | 0,00 | 0,00 |
| unorganized_cluster | 0,01 | 0,98 | 0,00 | -0,01 | 0,01 | 0,00 |
| sloppy_cluster | 0,00 | 0,58 | 0,00 | 0,04 | 0,03 | -0,01 |
| unreliable_cluster | 0,01 | 0,54 | -0,04 | -0,04 | -0,01 | -0,02 |
| bureaucratic_cluster | 0,01 | 0,00 | 0,97 | 0,00 | -0,02 | 0,00 |
| matrixed_cluster | 0,00 | 0,04 | 0,90 | -0,01 | 0,01 | 0,00 |
| hierarchical_cluster | 0,02 | -0,01 | 0,86 | -0,02 | 0,00 | 0,01 |
| political_cluster | 0,11 | -0,11 | 0,56 | 0,08 | 0,02 | 0,00 |
| ambiguous_cluster | 0,01 | 0,01 | 0,00 | 0,97 | -0,01 | -0,02 |
| vague_cluster | 0,00 | -0,01 | 0,00 | 0,97 | 0,00 | -0,01 |
| conflicting_cluster | 0,03 | 0,00 | 0,00 | 0,69 | 0,01 | 0,05 |
| contradictory_cluster | 0,04 | -0,02 | 0,01 | 0,52 | 0,05 | 0,07 |
| complicated_cluster | 0,02 | 0,00 | -0,01 | -0,02 | 0,99 | -0,01 |
| convoluted_cluster | 0,02 | 0,03 | -0,03 | 0,02 | 0,82 | 0,04 |
| complex_cluster | 0,03 | 0,01 | 0,08 | -0,03 | 0,71 | -0,05 |
| confusing_cluster | 0,02 | -0,04 | -0,04 | 0,04 | 0,67 | 0,02 |
| manipulative_cluster | 0,00 | 0,01 | 0,01 | -0,01 | -0,01 | 0,98 |
| untrustworthy_cluster | 0,00 | 0,01 | 0,01 | -0,01 | -0,01 | 0,98 |
| dishonest_cluster | 0,02 | -0,01 | -0,04 | 0,03 | 0,07 | 0,57 |
| deceptive_cluster | 0,01 | -0,05 | -0,07 | 0,04 | 0,02 | 0,56 |

## Section 4.2: Extracting example sentences

To create the list of example sentences that supported the naming of the clusters as described in the main article, I randomly extracted 25 sentences per cluster (31 clusters) with a fixed seed (seed = 42). The full list of example sentences is available in the OSF repository.
